# Measuring Quality of Life in Carers of People With Dementia: Development and Psychometric Evaluation of Scales measuring the Impact of DEmentia on CARers (SIDECAR)

**DOI:** 10.1093/geront/gnz136

**Published:** 2019-11-05

**Authors:** Mike C Horton, Jan Oyebode, Linda Clare, Molly Megson, Leanne Shearsmith, Carol Brayne, Paul Kind, Zoe Hoare, Hareth Al Janabi, Val Hewison, Alan Tennant, Penny Wright

**Affiliations:** 1 Psychometric Laboratory for Health Sciences, University of Leeds, UK; 2 Centre for Applied Dementia Studies, University of Bradford, UK; 3 Centre for Research in Ageing and Cognitive Health, University of Exeter, UK; 4 Leeds Institute of Medical Research at St James’s, University of Leeds, UK; 5 Cambridge Institute of Public Health, University of Cambridge, UK; 6 Leeds Institute of Health Sciences, University of Leeds, UK; 7 School of Health Sciences, Bangor University, UK; 8 Institute of Applied Health Research, University of Birmingham, UK; 9 Carers Leeds, UK

**Keywords:** Carers, Dementia, Quality of life, Needs-led, Rasch measurement, Psychometrics

## Abstract

**Background and Objectives:**

A 2008 European consensus on research outcome measures in dementia care concluded that measurement of carer quality of life (QoL) was limited. Three systematic reviews (2012, 2017, and 2018) of dementia carer outcome measures found existing instruments wanting. In 2017, recommendations were published for developing reliable measurement tools of carers’ needs for research and clinical application. The aim of this study was to develop a new instrument to measure the QoL of dementia carers (family/friends).

**Methods:**

Items were generated directly from carers following an inductive needs-led approach. Carers (*n* = 566) from 22 English and Welsh locations then completed the items and comparator measures at three time points. Rasch, factor, and psychometric (reliability, validity, responsiveness, and minimally important differences [MIDs]) analyses were undertaken.

**Results:**

Following factor analysis, the pool of 70 items was refined to three independent scales: primary SIDECAR-D (direct impact of caring upon carer QOL, 18 items), secondary SIDECAR-I (indirect impact, 10 items), and SIDECAR-S (support and information, 11 items). All three scales satisfy Rasch model assumptions. SIDECAR-D, I, S psychometrics: reliability (internal ≥ .70; test–retest ≥ .85); convergent validity (as hypothesized); responsiveness (effect sizes: D: moderate; I and S: small); MIDs (D = 9/100, I = 10/100, S = 11/100).

**Discussion and Implications:**

SIDECAR scales demonstrate robust measurement properties, meeting COSMIN quality standards for study design and psychometrics. SIDECAR provides a theoretically based needs-led QoL profile specifically for dementia carers. SIDECAR is free for use in public health, social care, and voluntary sector services, and not-for-profit organizations.

In 2017, the Alzheimer’s Association estimated that carers of people with dementia in the United States provided 18.4 billion hours of unpaid care annually, equating to a cost of $232.1 billion (Alzheimer’s Association, 2018). We define a “carer” as someone who is unpaid in supporting a friend or family member with dementia who cannot manage without their assistance. These many hours of unpaid care protect society from a huge financial burden but possibly at considerable personal carer cost. Although some carers report positive outcomes, many report negative impacts on their quality of life (QoL) (Alzheimer’s Association, 2018). Reasons for diminished carer QoL are complex (Sörensen & Conwell, 2011), and it has been recognized that the physical and mental health needs of carers should be tracked in order to limit preventable carer burden (Khachaturian et al., 2017). Evaluating the effectiveness of help, in terms of carer benefit and value for money, is a responsibility of service providers and clinical trialists. One method either to identify carers at risk of reduced QoL or to measure intervention effectiveness is to assess carer QoL. Many approaches have been taken to assess dementia carer outcomes with a multitude of existing, and overlapping generic, carer-specific and dementia carer-specific questionnaires employed used in clinical/social care practice, clinical trials, service evaluation, and economic evaluation (Moniz-Cook et al., 2008; Mosquera et al., 2016). In their systematic review of measures of the impact of caring for an elderly relative, Mosquera and coworkers (2016) conceptualize QoL and caregiver burden as two major constructs that represent the highest levels of integration of domains of impact, as opposed to scales that integrate a narrower range, such as questionnaires on impact of caregiving on physical health or psychosocial functioning. They suggest that QoL is a more generic construct while caregiver burden is more specific. QoL is essentially a positive construct which brings together a range of dimensions that, when fulfilled, constitute “the good life” (Lawton, 1983, p. 349), in contrast to burden which reflects the impact of stress and strain. This connects well with the current emphasis on positive psychology and well-being (Seligman, 2018) and recognition of the need to research the positive as well as the negative aspects of caregiving (Quinn et al., 2019).

Three systematic reviews of QoL questionnaires for use with dementia carers have been completed, but these reviews reached no consensus about which questionnaire, if any, delivers the measurement standards that are required across both descriptive and scoring/valuation systems (Dow et al., 2018; Jones, Edwards, & Hounsome, 2012; Page et al., 2017). The World Health Organization defines QoL as multidimensional including physical, psychological, and social domains, as a minimum (Kuyken et al., 1995). However, carers may be physically and psychologically well and have no functional limitations but be severely restricted in their everyday lives directly or indirectly by their caring responsibilities. It is therefore challenging to identify a QoL questionnaire that is relevant to dementia carers, meets measurement standards, and is “fit for purpose” for varied objectives.

One approach to measuring QoL originates from a “needs-based” theoretical premise, where: “Life gains its quality from the ability and capacity of the individual to satisfy his or her needs,” with QoL high when needs are fulfilled and low when few needs are fulfilled (Hunt & McKenna, 1992, p. 307). This model is therefore conceptually unidimensional, meaning all items (questions) reflect a single underlying latent trait. Questionnaire content is derived “bottom-up” from the “client group” only, rather than from a professionally driven agenda (Doward, McKenna, & Meads, 2004). The needs-led approach focuses on fundamental human needs (e.g., need for affection, for freedom) (Maslow, 1943) rather than more external or service-related needs (e.g., for information, for services) (McCabe, You, & Tatangelo, 2016). This approach may be particularly relevant to carers of people with dementia. A model, based on the needs-led premise, delineates the direct relationship between needs fulfilment and QoL in people with dementia (Scholzel-Dorenbos, Meeuwsen, & Olde Rikkert, 2010). This approach has been recommended in developing QoL questionnaires for carers of people with dementia (Bangerter, Griffin, Zarit, & Havyer, 2019).

The subject of this paper is the development and psychometric evaluation of the new QoL questionnaire known as SIDECAR (Scales measuring the Impact of DEmentia on CARers). A heath economic valuation of SIDECAR will be published separately.

## Methods

### Item Generation and Response Format

Item generation, reported in detail elsewhere (Oyebode et al., 2018; Pini et al., 2018), comprised interviews, to capture the impact of caring, with 42 carers of a relative with dementia (Alzheimer’s disease, vascular dementia, other forms of dementia) living in the community. Where possible, exact phrases provided the wording for the initial 99 items generated. These were subject to checks regarding ambiguity, content, and face validity. Twenty-two cognitive interviews with carers pretested and assessed response formats. Final review and an administration rehearsal with two carers resulted in an item pool of 70 dichotomous (agree/disagree) items, some positively and others negatively phrased (Oyebode et al., 2018).

### Psychometric Testing

COnsensus-based Standards for the selection of health Measurement Instruments (COSMIN: https://www.cosmin.nl/) were followed (Mokkink et al., 2010). Ethical approval was awarded by the Health Research Authority South West - Exeter Research Ethics Committee (16/SW/0280).

### Study Design

Participants were invited to complete a questionnaire pack at three time points: Time 1 (T1), following consent; Time 2 (T2), 2–4 weeks later; and Time 3 (T3), for a subsample (due to time constraints), 6 months after Time 1.

### Participants

Participants were English-literate primary carers, at least 16 years of age, supporting a partner or family member with a diagnosis of dementia living in the community. Twenty-two clinical network teams in England and Wales recruited carers via health and social care services (e.g., memory clinics); third-sector organizations (e.g., charities); the National Institute for Health Research Join Dementia Research (JDR: https://www.joindementiaresearch.nihr.ac.uk/) database (Juaristi & Dening, 2016); and carers involved in the “IDEAL” study (Improving the Experience of Dementia and Enhancing Active Life) at the time of their third interview (Clare et al., 2014).

### Study Measures

#### SIDECAR item pool (T1, T2, and T3)

This comprises 70 short statement items generated from the qualitative interviews such as, “I have had to put my own life on hold,” with response options: “agree”/“disagree” (Oyebode et al., 2018). The time frame relates to “today.”

#### Short Warwick–Edinburgh Mental Well-being Scale (T1 and T3)

Short Warwick–Edinburgh Mental Well-being Scale (SWEMWBS) is the shortened seven-item scale derived from the Warwick–Edinburgh Mental Well-being Scale (WEMWBS) (Stewart-Brown et al., 2009). The positively worded items cover feeling and functioning aspects of mental well-being over the preceding 2 weeks, for example: “I’ve been feeling useful.” Each item has five response categories (“none of the time” through to “all of the time”) which, when summed, create a score ranging from 7 to 35, with higher scores denoting higher well-being.

#### EuroQol Group EQ-5D 3L (T1 and T3)

The EQ-5D 3L is a five-item measure which assesses mobility, self-care, usual activities, pain/discomfort, and anxiety/depression, with a three-option response format, (“no problems,” “some problems,” and “severe problems”—EuroQol Group, 1990). The EQ-5D 3L includes a Visual Analogue Scale, rated from “0” (worst imaginable health state) to “100” (best imaginable health state) for “health state today.”

#### Sociodemographic details

At T1, carers provided sociodemographic details (e.g., age, sex, relationship to the cared for person) and information about the person cared for (e.g., age, dementia diagnosis). At T2 and T3, carers reported whether their caring situation had changed (better or worse) or remained the same since the last time point. At T3, carers rated any change in their overall QoL in the last 6 months using a 5-point response option (“Much worse,” “Worse,” “About the same,” “Better,” and “Much better”).

### Sample Size

A sample size of 400 was targeted, based on Rasch analysis requirements to provide stable item calibrations (Linacre, 1994), and avoid Type I errors (Hagell & Westergren, 2016).

### Scale Development

Participants with more than 90% item pool responses missing were excluded (*n* = 4). Across all other participants (*n* = 566), the mean amount of missing responses across the 70-item pool was 1 (*SD* 4.28; median 0 [interquartile range 0–0]), with 80% of participants having complete data. For factor analysis and Rasch analysis, all available data at T1 were used, without imputation.

#### Exploratory factor analysis

Earlier work indicated that the item pool covered several themes (Pini et al., 2018), suggesting the complete item set may not lend itself to measuring a single overarching construct. Therefore, preliminary exploratory factor analysis (EFA) was undertaken on the item pool using MPlus 7.4 (Muthén & Muthén, 1998–2017). A tetrachoric correlation matrix and GEOMIN rotation were used to account for the ordinal nature of the data, with indicators of model fit provided by the root mean square error of approximation (RMSEA), comparative fit index (CFI), and Tucker–Lewis index (TLI). EFA identified item sets/factors (of different constructs) that were taken forward for further refinement through Rasch analysis.

#### Rasch analysis

The Rasch model is a unidimensional measurement model that satisfies the assumptions of fundamental measurement (Luce & Tukey, 1964; Newby, Conner, Grant, & Bunderson, 2009), meaning it provides a measurement template against which scales can be tested. Essentially, Rasch Measurement Theory (RMT) provides a way to assess multi-item latent scales to ensure it is valid to add the items together to form an overall total score. The application of RMT provides a unified confirmatory framework for several aspects of internal construct validity to be assessed, highlighting measurement anomalies within an item set.

Rasch analysis was completed with RUMM2030 software (Andrich, Sheridan, & Luo, 2010). All items were assessed for: individual fit to the Rasch model, relative to the item set, to test whether each item was contributing to the same underlying construct (nonsignificant at Bonferroni-adjusted chi-squared *p*-value, standardized [*z*-score] fit-residuals within ±2.5); local dependency, to determine whether the response to any item has a direct impact on the response to any other item (Q3 criterion cut point = .2 above average residual correlation—Christensen, Makransky, & Horton, 2017); item bias, in the form of uniform and non-uniform differential item functioning (DIF) by age, gender, and carer relationship (spouse/other) (nonsignificant at Bonferroni-adjusted analysis of variance *p*-value); and scale targeting (relative distribution of item and person locations) (Hagquist, Bruce, & Gustavsson, 2009). Additionally, a series of *t* tests was used to assess the unidimensionality assumption (Smith, 2002), where evidence of unidimensionality is apparent when independent subsets of items deliver significantly different person estimates, and the lower bound 95% confidence interval (CI) percentage of significantly different *t* tests is <5%.

When the assumptions of the Rasch model are satisfied, the sufficiency of the raw score allows for a linear, interval-level transformation of scores (Tennant & Conaghan, 2007). For all individuals, raw SIDECAR scale scores correspond with an interval-level logit value which was extracted from the Rasch analysis software. The linear logit values were subsequently converted into 0–100 scale values in order to aid interpretability.

### Psychometric Evaluation

Basic descriptive statistics were run for each scale, including floor and ceiling effects.

Internal Consistency and Convergent Validity were assessed using T1 data, which provides the largest available sample of independent responses.

#### Internal consistency reliability (T1 data)

This was assessed using Cronbach’s alpha, in addition to a Person Separation Index (PSI), derived from the Rasch analysis. The PSI should be interpreted in a similar way to Cronbach’s alpha, but it uses the Rasch-derived linear scores rather than raw scores, and it also takes into account the relative targeting of the scale. A minimum alpha value of .7 was set (Nunnally & Bernstein, 1994). Cronbach’s alpha values are only available when calculated for cases with complete data.

#### Test–retest reliability (T1 and T2 data)

Responses from participants recruited from March 2017 to study close, who returned the T2 survey within 6 weeks of the original survey and with “no change” in their caring situation, were included.

Test–retest reliability was calculated at item level using kappa, with interpretation of levels of agreement as follows: ≤ .20 poor, .21– .40 fair, .41– .60 moderate, .61– .80 good, and .81–1.00 very good (Altman, 1991). SIDECAR scales’ test–retest reliability was undertaken using the intraclass correlation coefficient (ICC) on converted scale scores (0–100). ICCs (95% CIs) were calculated using a mean rating, absolute agreement, two-way mixed effects model, with reliability interpretation as follows: < .5 poor, .5– .75 moderate, .75– .9 good, and > .9 excellent (Koo & Li, 2017).

#### Convergent validity (T1 data)

Based on clinical experience, a negative correlation was hypothesized between SIDECAR scales and well-being (SWEMWBS) and to a lesser extent, with health valuation (EuroQol Group Visual Analogue Scale [EQ-5D VAS]). Spearman’s rank correlation assessed the strength and direction of these associations. COSMIN recommends the following guidance for interpretation of the correlation coefficients: correlations measuring a similar construct should be ≥ .50; correlations with instruments measuring related but dissimilar construct should be lower, that is, .30– .50; correlations with instruments measuring unrelated constructs should be < .30. Correlations defined previously should differ by a minimum of .10 (Mokkink et al., 2018).

#### Responsiveness (T1 and T3 data)

Responsiveness represents an instrument’s ability to detect changes over time, and minimally important difference (MID) provides meaningful interpretation from the carer perspective (Revicki et al., 2006). All responsiveness indicators were based on the converted 0–100 SIDECAR scores of those that responded at T3 (*n* = 173). A number of anchor-based measures of responsiveness were calculated based on a self-reported worsening in QoL status between T1 and T3, pooling the groups stating their QoL was “worse” and “much worse” (*n* = 72; 41.6%). The responsiveness indicators reported are the effect size (ES), standardized response mean (SRM), responsiveness statistic (RS) (Revicki et al., 2006), and repeated measures effect size (RMES) (Morris & DeShon, 2002).

The MID was calculated relative to the group reporting no change in their QoL (*n* = 93; 53.8%) (Revicki et al., 2006). None of the responsiveness indicators are provided for the group reporting an improvement in self-reported QoL, due to insufficient numbers (*n* = 7; 4%).

In addition to the anchor-based indicators, smallest detectable difference (SDD) is a distribution-based indicator of responsiveness that was calculated based on the complete T1 sample.

## Results

### The Sample

#### Participants

The data reported are from 570 participants recruited between November 18, 2016 and December 7, 2017 (Table 1). Carer median age was 70 years (men: 75 years, range 36–92 years; women: 69 years, range 34–91 years). Most carers were of white ethnicity (97%).

**Table 1.** Sociodemographic Characteristics at T1

	*n*	%
Place of carer identification		
IDEAL	131	23.0
JDR	54	9.5
NHS service	240	42.1
Third-sector organizations	37	6.5
Other	50	8.8
Missing	58	10.2
Carer sex		
Men	151	26.6
Women	412	72.3
Missing	7	1.2
Relationship to the person being cared for		
Spouse or partner	424	74.4
Son or daughter	108	18.9
Son-in-law or daughter-in-law	11	1.9
Other (relative/ friend)	19	3.4
Missing	8	1.4
Left school at minimum school leaving age		
Yes	215	37.7
No	348	61.1
Missing	7	1.2
Gave up work to care for the person with dementia		
Yes	82	14.4
No	480	84.2
Missing	8	1.4
Employment status		
Not in paid work	465	81.6
Paid work (≥30 hr per week)	45	7.9
Paid work (<30 hr per week)	50	8.8
Missing	10	1.8
Living with the person being cared for		
Yes	461	80.9
No	102	17.9
Missing	7	1.2
Dementia severity of the person being cared for		
Mild	209	36.7
Moderate	271	47.5
Severe	74	13.0
Missing	16	2.8

Note: IDEAL = the Improving the Experience of Dementia and Enhancing Active Life Study; JDR = Join Dementia Research database; NHS = National Health Service. Dementia severity: Mild: needs some assistance with day-to-day life due to dementia but is still quite independent. Moderate: Has obvious difficulties with memory or thinking due to dementia and needs a lot of assistance with day-to-day life. Severe: Has great difficulty communicating and needs help with many aspects of personal care (e.g., washing, getting dressed, and eating).

At T1, *n* = 570 (566 valid); at T2, *n* = 100 (100 valid); at T3, *n* = 173 (172 valid).

#### Missing values

Four participants were excluded due to void responses. Excluding these participants (*n* = 566), all items had ≤3% missing values excepting one: “Receiving help is more hassle than it’s worth” (6.7% missing).

### SIDECAR Scales Development

#### Rasch analyses and factor analysis

**Figure 1.**
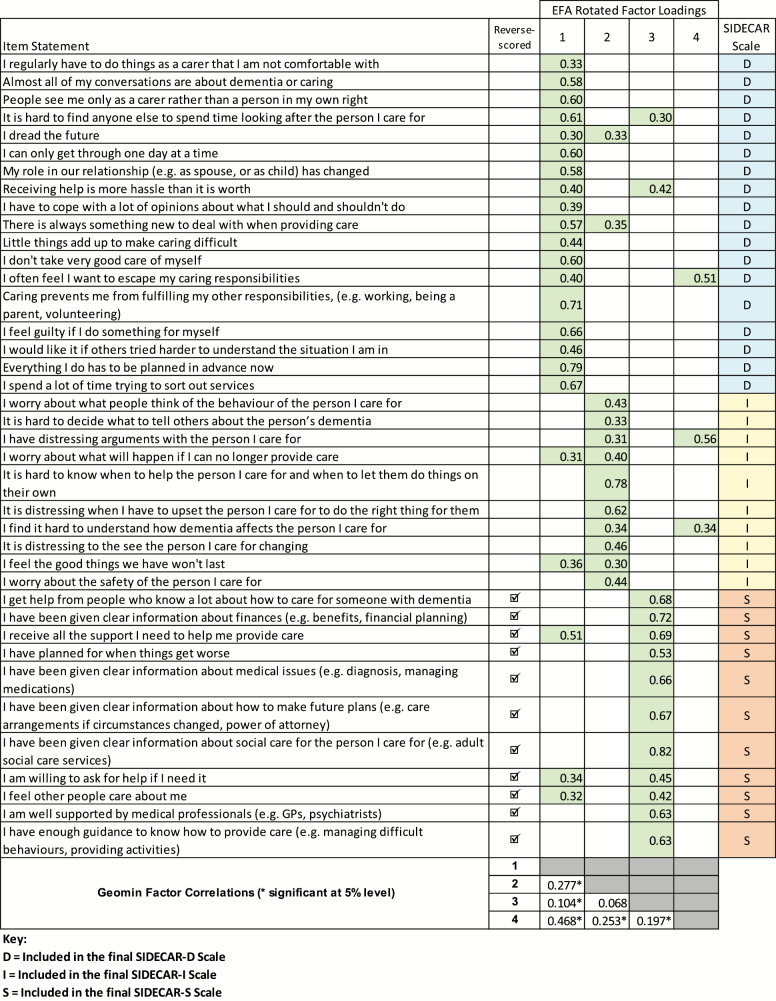
EFA Rotated Factor Loadings From 70-Item Set, Reported for the 39 Items Retained in Final SIDECAR Scales.Note: EFA = exploratory factor analysis; GP = general practitioner. D = included in the final SIDECAR-D scale; I = included in the final SIDECAR-I scale; S = included in the final SIDECAR-S scale.

Initial Rasch analysis of the 70-item set revealed extensive misfit and severe breach of the unidimensionality assumption, with a series of *t* tests reporting significantly different person estimates in 29% (lower CI = 27%) of cases (Table 2), suggesting a multidimensional item set. EFA identified four potential factors (RMSEA = .021, CFI = .966, TLI = 0.962) (Figure 1 and Supplementary Figure S1).

**Table 2.** Rasch Analysis Summary Statistics of SIDECAR Scales

	Analysis	Number of items	valid *n* (no. of extremes)	Item fit residual		Person fit residual		Overall chi-squared interaction					Unidimensionality t tests (CI)	
				Mean	*SD*	Mean	*SD*	Value	df	*p*	PSI	Alpha	Proportion significant	CI
Complete item set	Initial	70 items	566 (0)	−0.16	2.64	−0.15	0.92	1229.8	560	<.0001	.92	.92	.29	.27–.31
SIDECAR-D	Initial	37 items	558 (8)	−0.4	2.13	−0.17	0.92	558.8	296	<.0001	.9	.91	.12	.10–.14
	Final	18 items	547 (18)	−0.32	1.13	−0.2	0.72	184.1	162	.11	.81	.83	.04	.02–.06^a^
SIDECAR-I	Initial	12 items	532 (34)	−0.48	1.21	−0.18	0.64	123.3	84	.003	.66	.75	.02	.00–.04^b^
	Final	10 items	513 (53)	−0.4	1.06	−0.19	0.59	79.7	60	.046	.58	.7	.01	.00–.03^b^
SIDECAR-S	Initial	14 items	511 (54)	−0.61	2.21	−0.1	0.8	210.6	98	<.0001	.75	.85	.04	.02–.06^b^
	Final	11 items	503 (61)	−0.45	1.39	−0.12	0.79	138.3	88	<.001	.69	.81	.01	.00–.03^b^
	Target values			0	1	0	1	Nonsignificant			>0.7	>.7	Lower CI < .05	

*Note*: CI = confidence interval; df = degrees of freedom; PSI = Person Separation Index. Extremes = people scoring either maximally or minimally across the complete item set.

^a^Limited power in unidimensionality *t* test.

^b^Low power in unidimensionality *t* test.

Rasch analysis of the four factors revealed varying levels of overall fit, and a number of individual misfit anomalies (Table 2). Within each factor, scale refinement was conducted iteratively, where item misfit anomalies were identified and dealt with in order of their magnitude. Items displaying more than one aspect of misfit were selected as prime candidates for removal. This was undertaken in turn for all four factors starting with the first factor.

**Figure 2.**
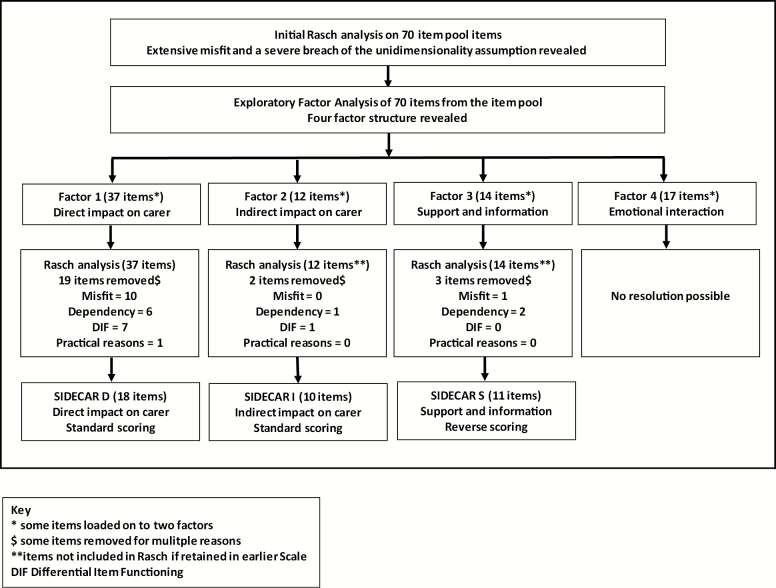
SIDECAR scale development process summary.

This process resulted in one primary scale of 18 items (SIDECAR-D), measuring the direct impact of caring on the carer and representing the a priori concept that “life gains its quality from the ability and capacity of the individual to satisfy their needs.” Two secondary scales were derived which reflect aspects of carer QoL that are more dependent on external circumstances (Figure 1; Figure 2). One of these reflects the status/circumstance of the person being cared for and how that affects the carer, and has therefore been labeled as measuring aspects of “indirect impact of caring” (SIDECAR-I; 10 items). The third scale, “support and information” (SIDECAR-S; 11 items), largely concerns more practical external support and feels distinctly different to the other two scales, demonstrated by the positive wording. No resolution was possible for the fourth factor, which was also more conceptually ambiguous.

Within each factor, when the final refined item set had been configured, each of the removed items was individually added back into the final item set, in order to test whether the original source of misfit (and reason for removal) remained. This was the case for all removed items, thus precluding any reintroductions. Please see Supplementary Figure S1 for items that were not retained in the final SIDECAR scales.

SIDECAR-D satisfies all assumptions of the Rasch model, at both the individual-item level and the scale level, indicating a unidimensional, psychometrically robust scale. SIDECARs I and S mostly satisfy the Rasch model assumptions, but some potential borderline issues remain. However, these supplementary scales contain useful information and are satisfactorily robust (see Table 2).

### Psychometric Evaluation

Descriptive statistics are provided in Table 3. Score distribution was good across all scales, with no evidence of significant floor or ceiling effects. All scales demonstrated acceptable internal consistency.

**Table 3.** Psychometric Properties of SIDECAR Scales

Scale property	Statistic	SIDECAR-D	SIDECAR-I	SIDECAR-S
Scoring (higher score represents worse QoL)	–	Negative	Negative	Positive
Number of items	–	18	10	11
Raw score range	–	0–18	0–10	0–11
Number of distinct measurement points	–	19	11	12
% missing responses per item	Range	0–6.7%	0.2–1.6%	0.7–3.0%
Descriptive statistics	Mean (*SD*)	46.66 (18.12)	68.00 (18.77)	41.68 (23.81)
	Median	47.25	70.07	38.96
	Skewness	−0.21	−0.49	0.12
	Kurtosis	0.21	0.62	−0.5
Ceiling effect (100)	Count (%)	2/565 (0.4%)	49/566 (8.7%)	11/564 (2.0%)
Floor effect (0)	Count (%)	16/565 (2.8%)	4/566 (0.7%)	50/564 (8.9%)
Internal reliability	Cronbach’s alpha	.83	.7	.81
Test–retest reliability^a^	ICC (95% CI)	.86 (.80−.91)	.86 (.80−.91)	.85 (.78−.90)
Convergent validity (SWEMWBS)	Spearman’s *R*	−.57	−.4	−.36
Convergent validity (EQ-5D VAS)		−.35	−.21	−.24
SIDECAR scale I intercorrelation	Pearson’s *R*	.55	–	–
SIDECAR scale S intercorrelation		.29	.25	–

*Note*: CI = confidence interval; EQ-5D VAS = EuroQol Group Visual Analogue Scale; ICC = intraclass correlation; QoL = quality of life; SWEMWBS = Short Warwick–Edinburgh Mental Well-being Scale. All scale scores based on converted 0–100 values of Time 1 sample (*n* = 566).

^a^Test–retest carried out Time 1–Time 2 sample (*n* = 100).

#### Test–retest reliability

Responses from 100 carers met inclusion criteria. Two items had “fair” kappa values ( .38; .40), the rest were moderate or good (excepting one where kappa was not calculated as all participants “agreed” at T1). SIDECAR scales demonstrated very good overall test–retest reliability.

#### Convergent validity

Convergent validity was supported with all scales negatively correlated more highly with SWEMWBS than with EQ-5D VAS, as hypothesized. All differences between the correlation coefficients of the SIDECAR scales with the two measures were greater than .10.

#### Responsiveness

RS is provided in Table 4. SIDECAR-D demonstrated a moderate responsiveness ES, with the supplementary SIDECARs I and S demonstrating a small ES. Using the higher 95% CI for the MIDs to indicate a worsening in QoL (a higher score), the MID values are 8.71, 9.73, and 10.96 for SIDECAR-D, I, and S, respectively (on a 0–100 linear scale). These values represent the score shift that is meaningful from the carer’s perspective.

**Table 4.** Anchor-Based Scale Responsiveness of SIDECAR Scales for Those Indicating Worse Quality of Life at T3 Compared With T1

Statistic	SIDECAR-D		SIDECAR-I		SIDECAR-S	
Self-reported QoL status	Worse	Stable	Worse	Stable	Worse	Stable
*n*	72	93	72	93	72	93
T1 mean	53	42.09	73.41	66.12	42.25	39.52
T1 SD	14.3	17.46	17.65	18.14	25.64	21.93
T3 mean	59.18	43.35	78.56	66.19	45.09	37.17
T3 SD	14.19	17.65	17.79	18.92	26.28	22.89
T3 mean–T1 mean	6.18	1.25	5.15	0.08	2.84	-2.35
*SD* of change	10.97	13.6	14.93	15.13	17.7	19.72
ES	0.43		0.29		0.11	
SRM	0.56		0.34		0.16	
RS	0.45		0.34		0.14	
RMES	0.56		0.35		0.16	
MID (95% CI)	4.93 (1.15–8.71)		5.07 (0.42–9.73)		5.19 (-0.58–10.96)	
SDD^a^	20.71		28.5		28.77	

*Note*: CI = confidence interval; ES = effect size; QoL = quality of life; MID = minimally important difference; RMES = repeated measures effect size; RS = responsiveness statistic; SDD = smallest detectable difference; SRM = standardized response mean; T1 = Time 1; T3 = Time 3. All scale scores based on converted 0–100 values.

^a^SDD is a distribution-based responsiveness indicator, calculated on T1 data only.

The items included in the three SIDECAR scales are indicated in Figure 1, and the final scales and scoring algorithms are available via (www.licensing.leeds.ac.uk).

## Discussion and Implications

We have described the development and psychometric evaluation of SIDECAR-D, I, and S, a questionnaire designed to evaluate the QoL of carers of people with dementia for use in clinical/social care practice, research, and service evaluation. The research has met recognized international criteria set by COSMIN in terms of not only the quality of the study, but also the psychometric properties reported (Mokkink et al., 2010). In line with other “needs-led” QoL questionnaires (Doward et al., 2004), a higher score indicates poorer QoL, reflecting the increasing impact of caring for someone with dementia. In this respect, the questionnaire has considerable overlap with scales of carer burden. If high, burden would be expected to impact negatively on QoL. A comparison of items of SIDECAR with those in the 22-item Zarit Burden Interview (ZBI) (Zarit, Orr, & Zarit, 1987, pp. 83–85) indicates that six items from the two scales are very similar (e.g., “I often feel I want to escape my caring responsibilities.” [SIDECAR] cf “Do you wish you could leave the care of your relative to someone else?” [ZBI]). The remaining items of the ZBI tap subjective impact of a range of issues mostly with direct reference to the person who is cared for (e.g., Do you feel you should be doing more for your relative?) whereas the items in SIDECAR-D, in particular, refer back to the carer (e.g., I feel guilty if I do something for myself.) In this respect, this difference reflects that described by Mosquera and coworkers (2016) of burden being more specific and QoL being more generic.

Although the primary focus was on QoL derived from fundamental universal human needs (Hunt & McKenna, 1992), our study has resulted in three SIDECAR scales reflecting differing needs-led QoL domains. SIDECAR-D arises directly from universal human needs, whereas SIDECAR-I reflects a more indirect impact of caring on QoL, and SIDECAR-S has a more external focus on support and information needs. SIDECAR scales may be used independently, or alongside each other to provide a profile of QoL across these domains.

It has been recognized that the social impact of the continuing increase in dementia prevalence will be ongoing (Khachaturian et al., 2017). The well-being of family carers is paramount to prevent further escalation of the issue, and therefore relevant measurement tools are necessary to monitor carer QoL. The universality of the needs-based model may provide the basis for generalized measurement, enabling international comparisons.

Needs-based QoL scales have been created for a variety of specific patient groups, for example with Crohn’s disease (Wilburn, McKenna, Twiss, Kemp, & Campbell, 2015) and ankylosing spondylitis (Doward et al., 2003). A more generic needs-led questionnaire, the CASP-19, is used widely in studies of early old age (Hyde, Higgs, Wiggins, & Blane, 2015; Hyde, Wiggins, Higgs, & Blane, 2003). Recently, a specific questionnaire for spouses of people with Alzheimer’s Disease was robustly developed and evaluated. Although it was initially intended for all family carers of those with Alzheimer’s disease, the psychometric evaluation did not support wider family application; it thus is restricted to spouses/partner carers of those with Alzheimer’s disease (Hagell, Rouse, & McKenna, 2018).

The rigorous conceptual and psychometric development of the SIDECAR scales demonstrates that they are all robust with wide application potential. The item reduction process ensured (within each scale) all items relate to the same construct (unidimensionality), and are statistically independent, thus validating a total scale score. Also, items are free from item bias (DIF) by age, gender, and carer relationship, meaning the scales operate equivalently across different types of informal carer (e.g., partner or child; male or female). All scales are appropriately targeted, meaning the scales cover the measurement range of carer QoL we wish to capture, with a minimal floor or ceiling effects. The fourth potential factor, which did not stand up to the rigorous standards demanded, contained items that were broadly associated with the emotional interaction between the person with dementia and their carer, but there was conceptual ambiguity within the item set, along with associated psychometric issues.

All SIDECAR scales performed well in psychometric tests, with SIDECAR-D demonstrating the strongest properties overall. SIDECAR scales exhibited “good” to “very good” internal and test–retest reliability. Confirmation of content and face validity was undertaken in the item generation phase of SIDECAR development (Oyebode et al., 2018). Establishing convergent validity requires relevant measures to be available for comparative purposes. Our hypothesis that “SIDECAR scales would be more closely correlated with well-being than health-related QoL” was substantiated.

The three reviews of QoL questionnaires for use with dementia carers highlighted the absence of responsiveness testing (Dow et al., 2018; Jones et al., 2012; Page et al., 2017), which can be reported in various ways (Mokkink et al., 2010; Revicki et al., 2006). One exception reported in the Dow and coworkers’ (2018) review was the Caregiver Quality of Life Instrument (Mohide, Torrance, Streiner, Pringle, & Gilbert, 1988), but this was tested with nine carers only. Responsiveness evaluation was anchored on self-reported change in QoL over the preceding 6 months of carers as no objective external “gold-standard” was available. We demonstrated that all SIDECAR scales detected changes in QoL over time, although the ES for SIDECAR I and S were small. Using the same self-reported QoL anchor, MIDs were established for the 0–100 Rasch-converted linear scores. However, there was no single MID value applicable across all populations and applications (Revicki et al., 2006), so this estimation should be repeated in future studies.

### Limitations

Although the initial item generation was based on a diverse sample (Oyebode et al., 2018), and the sociodemographic characteristics of the sample were broadly representative of informal carers of people with dementia in the United Kingdom (Alzheimer’s Research UK, 2018), there was under-representation of carers from minority ethnic groups. There are persisting barriers to use of mainstream dementia services by minority ethnic communities in the United Kingdom (Parveen, Peltier, & Oyebode, 2017). Assessment tools must be culturally sensitive to reduce any chance of measurement bias and to maximize inclusivity.

A point of debate during the item generation phase was the mix of positive and negative item phrasing (Oyebode et al., 2018). It was impossible to change the valence of some items and maintain the integrity of meaning conveyed by the carers, so item phrasing remained bi-directional. Although this was accounted for in the scoring of the items, the psychometric refinement resulted in one scale (SIDECAR-S) having all positively worded items, opposed to the two other negatively worded scales. Although it is not known whether this item set was identified purely due to the scoring direction, the content of the final SIDECAR-S item set suggests the items belong together conceptually.

Additionally, no gold-standard measure of carer QoL is currently available, so all responsiveness measures were based on carer self-assessment. Although self-assessment is an important and meaningful anchor, this should also be triangulated against other measures of change (Revicki et al., 2006).

### Further Studies

Prospective testing of the questionnaire is planned with new samples in different clinical and voluntary sector settings. This will evaluate usability and usefulness for clinical utility, service provision, individual carer assessment, and health economic functioning. Adoption into the IDEAL program (Clare et al., 2014; Silarova et al., 2018) will enable additional testing in a research context, allowing for the investigation of testable hypotheses of relationships between carer QoL and IDEAL study variables. Additional aims include to work towards the adoption of SIDECAR into the NHS Digital Indicator Governance Board library, to enable the impact of dementia on carers to be monitored at a national level, and to utilize SIDECAR within intervention trials, to gauge interventional impact on carers and to extend responsiveness testing.

### Conclusion

The SIDECAR scales were derived directly from carers and satisfy rigorous psychometric criteria. The primary scale (SIDECAR-D) is firmly grounded in the fulfilment of universal human needs. SIDECAR scales may be used independently, or alongside each other to provide a profile of QoL. The results indicate SIDECAR may be useful in individual carer assessment, or at group level in research and service evaluation. The raw score of each SIDECAR scale is valid as an ordinal unidimensional score, but the satisfaction of Rasch model assumptions also means that a 0–100 interval-level equivalent transformation is available for complete data. SIDECAR-D has been subjected to a valuation analysis, which will be separately reported.

SIDECAR is free for use in public health, social care, and voluntary sector services, and not-for-profit organizations. To use SIDECAR please register with the University of Leeds Fast Licensing Platform (www.licensing.leeds.ac.uk). The interview and questionnaire data are available via the University of Leeds data repository for academic purposes subject to request (https://doi.org/10.5518/433).

SIDECAR “© Copyright Universities of Bangor, Birmingham, Bradford, Cambridge, Exeter & Leeds.”

## Supplementary Material

Supplementary data are available at *The Gerontologist* online.

Supplementary Figure S1. EFA Rotated Factor Loadings from 70-item set, reported for the 31 items that were not retained in final SIDECAR scales.

gnz136_suppl_Supplementary_Figure_1

## Notes

Conflict of Interest

None reported.
