# Supplementary material for: Measuring Quality of Life in Carers of People With Dementia: Development and Psychometric Evaluation of Scales measuring the Impact of DEmentia on CARers (SIDECAR)
Source: Gerontologist. 2019 Nov 5;61(3):e1–e11. doi: 10.1093/geront/gnz136 (PMC8023371; doi:10.1093/geront/gnz136)
Supplement: gnz136_suppl_Supplementary_Figure_1 [file gnz136_suppl_supplementary_figure_1.docx]

**Supplementary Figure S1** EFA Rotated Factor Loadings from 70-item set, reported for the 31 items that were not retained in final SIDECAR scales
